# Supplementary material for: Patterns of Intron Gain and Loss in Fungi
Source: PLoS Biol. 2004 Nov 30;2(12):e422. doi: 10.1371/journal.pbio.0020422 (PMC532390; doi:10.1371/journal.pbio.0020422)
Supplement: Table S1 — Also available at http://genes.mit.edu/NielsenEtAl/. (4.3 MB ZIP). [file pbio.0020422.st001.zip › NielsenEtAl/html/1365.html]

AN1237.1.NCU02741.1.MG04832.1.FG01157.1


```
 CLUSTAL W (1.82) Multiple Sequence Alignments - Introns Inserted


Sequence 1: MG04832.1	353 aa
Sequence 2: FG01157.1	348 aa
Sequence 3: NCU02741.1	353 aa
Sequence 4: AN1237.1	344 aa
Alignment Length: 354 aa
Number Identitical Residues: 284 aa
Alignment Score (without introns) 11304


MG04832.1 	MTQVEDDYDESA-NADESGGAGPGAPTPLTALE0GVGGLTKRDIQLVMEGGYNTVESVAY
NCU02741.1	-MSNNEEYDESANMEDDSGIPNPGAPTPLSALE0GVAGLTKRDIQLIVDGGFNTVESVAY
FG01157.1 	---MSQEHDESQ-MGEEGGMPGPGAPTPLFALE0GVAGLTKRDIQLVVDGGYNTVESVAY
AN1237.1  	--------METQNEFDDSGLPGPGAPTPLSSLE0GMAGLTSRDIKLFVDAGYHTVESIAY
          	         *: .  ::.* ..******* :** *:.***.***:*.::.*::****:**

MG04832.1 	T~PRRMLEQIKGISEQKAGKILAEA1SKLVPMGFTTATEMHQRRSELISITTGSKNLDTM
NCU02741.1	T~PRRVLEQIKGISEQKAGKILAEA1SKLVPMGFTTATEMHQRRSELISITTGSKNLDTL
FG01157.1 	T~PRRVLEQIKGISEQKATKILAEA1SKLVPMGFTTATEMHQRRSELISITTGSKNLDTL
AN1237.1  	T2PKRLLEQIKGISEQKATKILVEA1AKLVPMGFTTATEMHARRSELISITTGSKQLDTL
          	* *:*:************ ***.** :************** *************:***:

MG04832.1 	LGGGIETGSVTELFGEFRTGKSQICHTLAVTCQLPFDMGGGEGKCLYIDTEGTFRPVRLL
NCU02741.1	LAGGIETGSVTEIFGEFRTGKSQICHTLAVTCQLPFDMGGGEGKCLYIDTEGTFRPVRLL
FG01157.1 	LAGGIETGSVTELFGEFRTGKSQICHTLAVTCQLPFDMGGGEGKCMYIDTEGTFRPVRLL
AN1237.1  	LGGGIETGSITEIFGEFRTGKSQICHTLAVTCQLPFDMGGGEGKCLYIDTEGTFRPVRLL
          	*.*******:**:********************************:**************

MG04832.1 	AVANRFGLSGEEVLDNVAYARAYNSDHQLQLLNQASAMMCETRFSLLIVDSATALYRTDF
NCU02741.1	AVANRYGLSGEEVLDNVAYARAYNSDHQLQLLNQAAAMMCETRFSLLIVDSATSLYRTDF
FG01157.1 	GVANRFGLSGEEVLDNVAYARAYNSDHQLQLLNQAAAMMCETRFSLLIVDSATSLYRTDF
AN1237.1  	AVAQRYGLVGEEVLDNVAYARAYNSDHQLQLLNQASQMMCETRFSLLVVDSATSLYRTDF
          	.**:*:** **************************: **********:*****:******

MG04832.1 	LGRGELSSRQTHLAKFMRQLQRLADEFGIAVVISNQVVAQVDGGPSAMFNPDPKKPIGGN
NCU02741.1	LGRGELSSRQTHLAKFMRTLQRLADEFGIAVVITNQVVAQVDGGPSAMFNPDPKKPIGGN
FG01157.1 	CGRGELSNRQTHLAKFMRTLQRLADEFGIAVVITNQVVAQVDGGPSAMFNPDPKKPIGGN
AN1237.1  	NGRGELSTRQTHLAKFMRTLQRLADEFGIAVVITNQVVAQVDGGPSAMFNPDPKKPIGGN
          	 ******.********** **************:**************************

MG04832.1 	IIAHASTTRLSLKKGRGETRIAKIYDSPCLPESDCLFAINEDGIGDPSPKDVEKMNN
NCU02741.1	IIAHASTTRISLKKGRGETRIAKIYDSPCLPESDCLFAINEDGIGDPSPKDMEKMNQ
FG01157.1 	IIAHASTTRISLKKGRAETRIAKIYDSPCLPESDTLFAIGEEGIGDPAPKDTEKD--
AN1237.1  	IIAHASTTRLSLKKGRGETRVCKIYDSPCLPESDCLFAINEDGIGDPSPKDLEND--
          	*********:******.***:.************ ****.*:*****:*** *:
```
